# Supplementary material for: Unifying the analysis of bottom-up proteomics data with CHIMERYS
Source: Nat Methods. 2025 Apr 22;22(5):1017–27. doi: 10.1038/s41592-025-02663-w (PMC12074992; doi:10.1038/s41592-025-02663-w)
Supplement: Supplementary file 1 — Supplementary Figs. 1 and 2, discussion, methods and references. [file 41592_2025_2663_MOESM1_ESM.pdf]

nature methods

Supplementary information https://doi.org/10.1038/s41592-025-02663-w

Unifying the analysis of bottom-up proteomics data with CHIMERYS

In the format provided by the authors and unedited

Supplementary Figures

Supplementary Figure 1 – Shared fragment ions

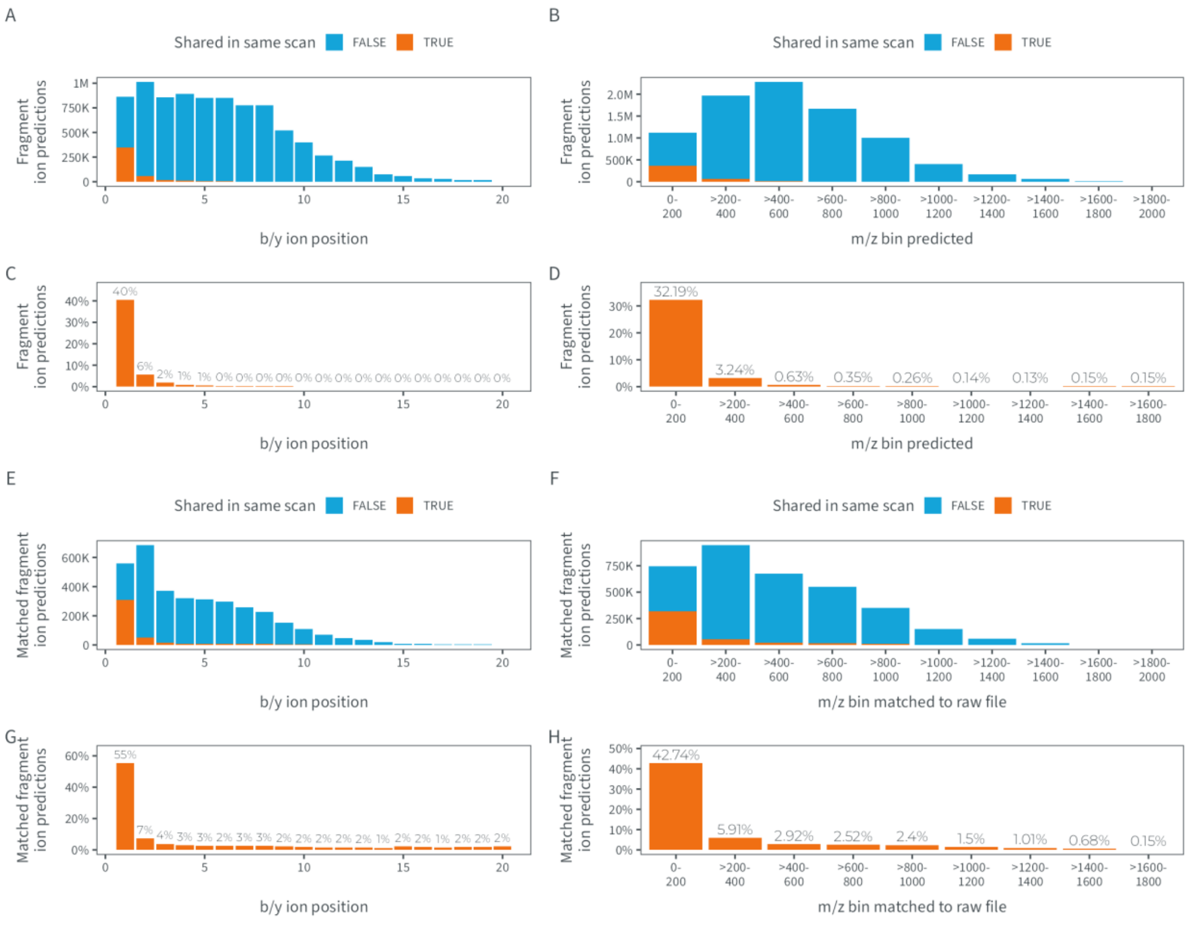

Absolute number **(A-B)** and relative fraction **(C-D)** of fragment ions shared (orange) and unshared (blue) between predicted spectra of PSMs. Fragment ions are shown as a function of fragment ion position **(A&C)** and fragment ion m/z **(B&D)**. Only PSMs that were identified by CHIMERYS at 1% run-specific PSM FDR in a 2-hour HeLa DDA single-shot measurement were considered (n = 1). Data was acquired on an Orbitrap QE HF-X with 1.3 Th isolation windows and taken from the LFQbench-type dataset[1]. **(E-F, G-H)** Analogous plots as in (A-B, C-D), but for shared (orange) and unshared (blue) fragment ions from predicted spectra that were matched to experimental peaks.

Supplementary Figure 2 – Chimeric DIA spectra

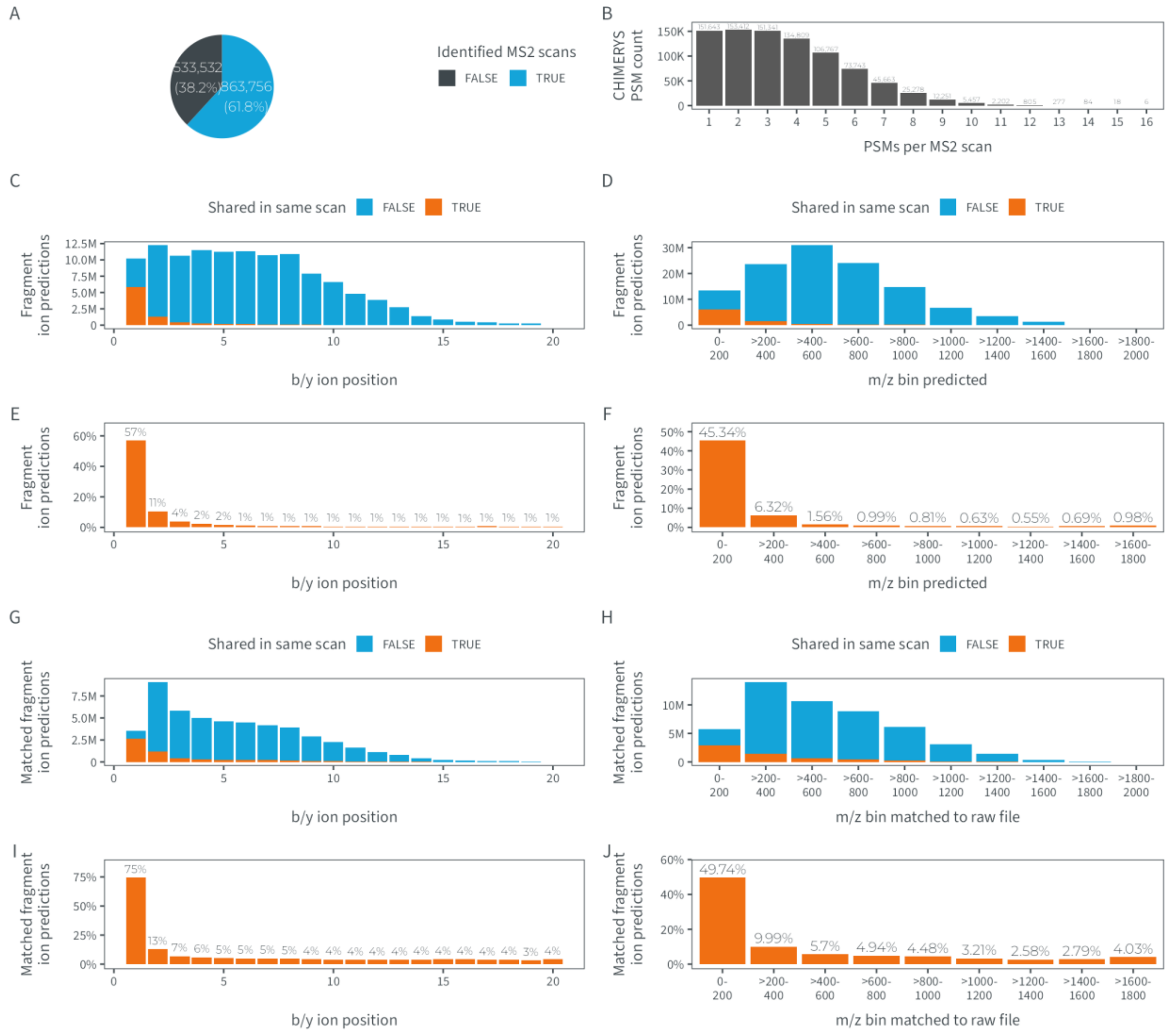

(A) Proportions of MS2 spectra with at least one confident (blue) or no confident PSM identification (gray) in triplicate 2-hour DIA single-shot measurements from two different conditions (n = 6). Data was acquired on an Orbitrap QE HF-X with 8 Th isolation windows and taken from the LFQbench-type dataset[1]. **(B)** Distribution of the number of PSMs per MS2 spectrum for the same data as in (A). **(C-F)** Absolute number **(C-D)** and relative fraction **(E-F)** of fragment ions shared (orange) and unshared (blue) between predicted spectra of PSMs. Fragment ions are shown as a function of fragment ion position **(C&E)** and fragment ion m/z **(D&F)**. Only PSMs that were identified by CHIMERYS at 1% run-specific PSM FDR from the same data as in (A) were considered. **(G-H, I-J)** Analogous plots as in (C-D, E-F), but for shared (orange) and unshared (blue) fragment ions from predicted spectra that were matched to experimental peaks.

Supplementary Discussion

We have demonstrated that our novel spectrum-centric search algorithm CHIMERYS is capable of deconvoluting chimeric spectra with accurate and sensitive PSM-level FDR control, irrespective of isolation window width, making it suitable for the analysis of DDA, DIA and PRM data alike. CHIMERYS is mindful of the fact that MS2 spectra can contain fragment ions from peptides the theoretical m/z of which falls into a corresponding isolation window, even if the precursor ion was not detected in the preceding MS1 spectrum. This frequently happens due to the limited dynamic range of MS1 spectra that acquire data across a large mass range and thus quickly reach the maximum number of ions that can be captured. MSFragger-DIA[2] takes a similar approach, however, its greedy algorithm that prevents experimental fragment ion peaks to contribute to the score of multiple different peptides is a subtraction approach, which limits its sensitivity, especially close to the detection limit, where typically only few fragment ions are matched, and some of which might be shared (e. g. b2- and y1-ions).

A disadvantage of CHIMERYS in the context of the limited dynamic range in MS2 spectra is its utilization of intensity-based scores such as the normalized spectral contrast angle (SA). As the intensity of a peptide drops, more and more of its low abundant fragment ions will disappear from the experimental spectrum. However, our machine learning model INFERYS – similar to most other machine learning models – was trained on high abundant peptide signals. As such, it will always predict the intensity of high abundant and low abundant fragment ions. When the latter are lost from an experimental spectrum under investigation because the peptide was low abundant, these peaks will remain unmatched. However, the SA will be calculated on matched (experimental and predicted intensity available), as well as on unmatched predicted peaks (only predicted intensity available). For the latter, the experimental intensity is set to zero, which reduces intensity-based scores such as the SA for low abundant peptides. This could be circumvented by performing intensity-dependent predictions, which we are currently exploring.

CHIMERYS' novel deconvolution-based approach allows it to distribute the experimental intensity of shared fragment ions to multiple peptides, proportional to their predicted contribution to the total ion current of the experimental spectrum. As a result, CHIMERYS maximizes the amount of explained experimental intensity with a minimal set of peptides. Merely ignoring low m/z regions to avoid shared fragment ions – as often done by DIA search engines – is not sufficient to protect against calling multiple identifications on the same signal. Additionally, CHIMERYS only reports the best scoring PSM per theoretical precursor mass for a given MS2 spectrum to further limit the number of highly similar (and isobaric) peptides being identified based on the same fragment ion information. In a way, this

approach returns to the roots of bottom-up proteomics, since CHIMERYS emphasizes the value of PSMs as the central information currency. Its deconvoluted spectra are tangible for the researcher as PSMs can be visually inspected using mirror plots. As it stands, CHIMERYS' acquisition method-agnostic data analysis approach requires highly accurate fragment ion intensities as obtained from pure peptide MS2 spectra, which is why predicted spectra are preferred over experimental spectral libraries, which themselves can contain chimeric spectra. Additionally, using predicted spectra as priors for peptide identification has the advantage that both target and decoy spectra can be predicted with equal quality, ensuring fair competition between targets and decoys. However, the requirement for predicted MS2 spectra is also a limitation of CHIMERYS, since it prevents its application to peptides carrying modifications that cannot yet be predicted by our deep learning model INFERYS. Some software packages that make use of predicted fragment ion intensities for the rescoring of PSMs such as MSBooster[3] circumvent this limitation by predicting the spectrum of the unmodified peptide and subsequently shifting the peaks corresponding to modified fragment ions without changing their predicted intensities. However, as already shown by the authors, the spectrum similiarity of these predictions to experimental data is considerably poorer than for peptides carrying PTMs the model they used was trained on. It is well known that certain PTMs substantially influence the fragmentation behaviour of peptides[4]. As a result, extrapolating to PTMs not present in the training dataset will result in poorer predictions for certain classes of peptides, which will have an impact on the likelihood of identifying them, thereby biasing results. For example, spectra of citrullinated peptides predicted with a model not trained on citrullination were shown to be similar to decoys, generating a 2-class-society of PSMs[4]. This is particularly detrimental for samples containing a mixture of peptide classes a given model was trained on and peptide classes it was not trained on, since rescoring software such as mokapot[5] will deprioritize features such as spectrum similarity, potentially limiting sensitivity for peptide classes with accurate predictions. In the future, this limitation should be lifted as deep learning models start to emerge that are capable of generalizing to previously unseen modifications, for example with the help of new peptide embeddings such as atomic compositions[6]. We are actively working on similar solutions that will allow INFERYS to extrapolate to previously unseen PTMs in the future.

We decided to set up a cluster of servers for predictions, rather than allowing them to be performed locally in order to reduce the runtime of CHIMERYS and facilitate supporting the software in the long-term. CHIMERYS needs to predict the MS2 spectrum for a given peptide up to twice per raw file: once for the first search and once – after collision energy (CE) recalibration – for the main search. This is because our deep learning model uses CE as an additional input, since it substantially influences fragmentation and can vary between mass

spectrometers and even over time for the same mass spectrometer[7]. However, in particular for large search spaces commonly encountered when working with dynamic modifications such as phosphorylation, predicting the entire spectral library up to twice per raw file is computationally expensive (2x >90M precursors per raw file as we need to predict multiple charge states). With ~2,000 predictions per second on a CPU and ~30,000 predictions per second on a GPU, this means that scientists would have to wait either 25h or 1.6h per raw file for these predictions to be performed using a single CPU or GPU, respectively. That is why we decided for GPU-based predictions in the cloud, where we can additionally scale out to multiple GPU-containing servers. This also allowed us to standardize and streamline the setup of these servers, which is quite important and often difficult, since successful predictions depend for example on matching GPU hardware and CUDA versions. In the future, we would like to also be able to perform predictions locally. However, for this to be feasible, current machine learning models need to become much faster on CPUs. INFERYS Rescoring[8] can run locally, because it only predicts the top 10 peptides per spectrum from Sequest HT, which drastically reduces the total number of predictions required.

As part of its deconvolution, CHIMERYS reports coefficients, which are spectrum-centric measures of peptide quantity and can effectively be interpreted as the interference-corrected total ion current of a given peptide. Other approaches typically select a fixed number of the most abundant fragment ions that do not suffer from interference (i. e. do not occur in more than one peptide) for quantification. This has the disadvantage that fragment ion interference can be highly sample specific, potentially resulting in different fragment ions being used for the quantification of the same peptide in different samples. Worst case, this can lead to wrong biological conclusions, especially when analyzing heterogeneous sample cohorts. Conversely, CHIMERYS' quantification considers all theoretical fragment ions that were used for identification also for quantification. We demonstrated that this novel quantification concept is precise and accurate even in the presence of shared fragment ions from co-eluting peptides, closely matches quantification via Skyline on PRM data and is highly correlated to quantification in MS1. As such, CHIMERYS is well-equipped for the analysis of heterogeneous sample cohorts such as population-scale plasma or cancer proteomics, as well as single-cell proteomics.

Nowadays, data on large and heterogeneous sample cohorts is often acquired using DIA, since it promises deep proteome coverage, high reproducibility and – most importantly – high data completeness given its systematic way of acquiring MS2 spectra. We have shown that entrapment experiments are important in order to avoid overestimating data completeness exemplified by DIA data analyzed with DIA-NN or Spectronaut. It is worth noting that we do not claim that all precursors not surviving eFDR thresholds are wrong identifications, but

rather that our entrapment experiments suggest that neither DIA-NN nor Spectronaut can claim them as confidently identified at 1% precursor-level FDR in the run-specific context.

Notably, Spectronaut quantified some precursors with low self-reported q-values but high eFDR and intensities close to zero. Inspecting fragment-level intensities of precursors identified by Spectronaut revealed that some fragment ions showed peak areas below 1 (Extended Data Figure 7C) and precursors with intensities close to 0 were quantified exclusively based on such fragment ions (Extended Data Figure 7D). Inspection of extracted ion chromatograms for such precursors – an example of which is shown in Figure 3D (bottom) – showed that they were quantified based on fragments with intensities between 0 and 1 across the putative elution of the precursor, which is in stark contrast to similarly high-scoring identifications shared between the three different search engines (Figure 3D top). Inspection of the corresponding raw data showed that these fragment ions sometimes had no signal at all at the relelvant retention time even though Spectronaut reported multiple data points per peak for the corresponding precursor, implying that Spectronaut performs some signal imputation even when imputation is explicitly turned off (Extended Data Figure 7E). We would argue that such fragment ions should be filtered out and also to remove precursors with fewer than three fragment ions used for quantification as initially configured in Spectronaut (Supplementary Methods).

Interestingly, Spectronaut behaved markedly differently when analyzed with the classic eFDR approach compared to the peptide and concatenated eFDR approaches (see Supplementary Methods). We hypothesize that this is due to the fact that entrapment identifications – unlike random false target identifications – are not randomly distributed across proteins using the classic eFDR approach, but rather always map to entrapment proteins, which – unlike proteins from the original target database – only contain false identifications. Therefore, entrapments generated with the classic eFDR approach can be trivially distinguished from random false target identifications by search engines that incorporate protein-level information into the precursor-level scores used for FDR estimation. An example of such a precursor-level score would be the average intensity of precursors that uniquely map to a given protein. Attaching such a score to each of the precursors mapping to the same protein will boost the confidence in true (non-random) and false (random) precursors mapping to true target proteins, because true precursor identifications will be based mostly on actual signal, while false precursor identifications will be based mostly on noise. Consequently, this would allow precursors mapping to true target proteins to score systematically higher than precursors mapping to entrapment proteins. If this happens during the generation of a spectral library, which is usually filtered to 1% FDR, sometimes at multiple levels, then there is a chance of removing entrapment precursors, but not random false target precursors from

said library. This discrepancy between random false target precursors and entrapment precursors is rectified with the concatenated eFDR and peptide eFDR approaches, which is desirable when evaluating precursor-level FDR estimates. As such, measurements of empirical precursor-level FDR with the concatenated eFDR and peptide eFDR approaches are more robust measurements of the true precursor-level FDR. DIA-NN seems to be anti-conservative in its FDR estimates, independent of the entrapment approach used. In fact, DIA-NN loses substantially more data completeness at peptide eFDR than CHIMERYS or Spectronaut. One explanation for this could be that the decoy library entries DIA-NN generates may be too dissimilar from target library entries. Notably, entrapments generated using mimic[9] can be very similar to targets, making it harder to distinguish them from each other, which is a desirable property for entrapments.

While a comprehensive evaluation of all DIA search algorithms is beyond the scope of this paper, we hypothesize that failure to control run-specific precursor-level FDR is due to the way spectral libraries are constructed in library-free workflows. Generating an FDR-filtered spectral library on DIA data and subsequently using it for the peptide-centric scoring of the same data could be considered double dipping, since information on the separation between targets and decoys is incorporated into the spectral library generation, which will in turn influence the peptide-centric FDR estimation performed later. As a result, the q-values of true and false peptide identifications could get boosted to a point where it is actually no longer possible to distinguish between true and false identifications. This would be particularly problematic if samples are highly heterogeneous. The more distinct the peptide set detectable in each sample, the more false identifications could potentially be introduced. Unfortunately, the widespread assumption that DIA measurements have high data completeness due to the way the data was acquired prompts researchers to expect very high data completeness (say 95% or higher), even when analyzing single cells, which are very heterogeneous almost by definition. This issue is exacerbated by novel mass spectrometers that drive sensitivity to the point of single ion detection. Recently, researchers began reporting record numbers of identifications on single cells with almost full data completeness. However, such data completeness claims need to be backed by strong evidence, otherwise, biological conclusions of individual experiments and the reputation of the entire DIA community are at risk.

In a maturing field of proteomics, where soon more biologists than MS experts utilize the technology for advancing research without inspecting the underlying raw data, software developers need to ensure that the output of their tools can be trusted and used at face value. This also creates the necessity to establish reporting guidelines for journals, minimal requirements for calling identifications (e.g. minimum number of matched fragment ions), standardized benchmark measures for evaluating search algorithms and clear community

standards for validating error control in DIA and other bottom-up proteomics data. Molecular and Cellular Proteomics has published such guidelines (https://www.mcponline.org/dia-guidelines), but these need to be overhauled to prevent a data reproducibility crisis in proteomics. The new entrapment approaches proposed herein are one contribution to managing quality and several more can be envisaged. Applying such tools will help software developers to meet the responsibility entrusted to them by their users.

In summary, we demonstrated that CHIMERYS properly controls the run-specific precursor-level FDR, while its deconvolution approach makes it agnostic to the chosen data acquisition method, which very timely ties into recent hardware developments, where smaller and smaller DIA windows are closing in on isolation widths typically used for DDA data acquisition. This calls for a harmonization of data analysis strategies, as the differences between DDA and DIA begin to go away[10]. The algorithm presented herein is well-equipped to be able to deal with any current and new acquisition methods and hence unifies the processing of bottom-up proteomics data.

Supplementary Methods

Training and fine-tuning INFERYS base models for peptide property prediction

Data preprocessing – fragmentation prediction

To train the INFERYS 3.0.0 base model for fragmentation prediction, publicly available data from various PRIDE identifiers were used as a training data foundation, notably the ProteomeTools project[11]. RAW data were downloaded and – where available – MaxQuant[12] search files were utilized. PSMs from MaxQuant's msms.txt were merged with the unprocessed scans extracted from RAW files with Thermo Fisher's Raw-FileReader (https://github.com/thermofisherlsms/RawFileReader). Data were filtered using various quality criteria, e.g. Andromeda score. The top 3 ranked PSMs by Andromeda score per sequence, charge, collision energy combination were selected across all files. For the PSMs, b- and y-ions, as well as several neutral losses (e.g. water, ammonia and carbon monoxide, phosphoric acid losses) were annotated for charge states 1-3. Amino acid tokens in the peptide sequence, precursor charge, and fragmentation type were one-hot encoded, and collision energy was normalized to [0, 1]. The data contains 25M PSMs and was split into 70% training, 20% test and 10% validation sets, ensuring that peptide sequences are not shared across splits.

Data preprocessing – retention time prediction

To train the INFERYS 3.0.0 base model for retention time prediction, the same unprocessed data was transformed similarly with the following differences: in addition to a stringent Andromeda score threshold, only the top-ranked PSMs by Andromeda score per sequence and file, and only the 5 top-ranked PSMs per sequence across all files were retained. Retention time was z-score normalized per file to account for differing run lengths. The resulting dataset contains 5.2M PSMs split into 70% training, 20% validation, 10% test sets, ensuring that peptide sequences are not shared across splits.

Model architecture

INFERYS 3.0.0 models for fragmentation and retention time prediction share a similar architecture. Sequences are processed with a PositionalEmbedding[13], a Transformer block and a GRU[14] layer (gated recurrent unit). For fragmentation prediction, additional meta information (precursor charge, collision energy and fragmentation type) is injected via a custom TransformerMixin layer to the sequence embedding outputs of the Transformer block (before the GRU layer). The TransformerMixin embeds one input parameter to the

dimensionality of a given Transformer output embedding and applies the product of the two to another Transformer layer. The final Transformer embedding is projected to the task-dependent output dimension (e.g. 1 output dimension for retention time). Models are built and trained with tensorflow 2.11.1.

Model training

The same training procedure was used for the fragmentation and retention time base model. Models were trained with the Adam optimizer[15] on the training split with early stopping, evaluating the validation split with patience 8 and a learning rate decay with a factor of 0.2 after 4 epochs without reduced validation loss for up to 200 epochs or till convergence. The retention time model optimized mean-absolute-error, and the fragmentation model optimized normalized spectral contrast distance[7,16]. Model hyperparameters, such as learning rate, batch size, dropout, embedding dimension, positional scaling, number of attention heads, and intermediate dimensions were optimized via Hyperband[17]. For the fragmentation model, we also optimized the order of meta parameter TransformerMixin layers via Hyperband.

Fine-tuning of the INFERYS base model for retention time prediction

During each CHIMERYS search, the INFERYS retention time base model is fine-tuned per raw file via refinement learning. Briefly, for each raw file, results of the first search are filtered to 1% FDR, followed by the removal of decoys, PSMs with a normalized spectral contrast angle smaller than 0.7 and PSMs for which the number of matched peaks divided by the number of predicted peaks is smaller than 0.6. Then the top-ranked PSM per spectrum by LDA score is selected and the top 10,000 PSMs by LDA score are split into training and validation set, ensuring that peptide sequences are not shared across splits. The retention time base model's layer weights are frozen except the last two dense layers, which are re-initialized and then fine-tuned on the refinement dataset. This results in distinct retention time models for each raw file that minimize the error to the corresponding experimental data, which effectively obviates the need for retention time alignment. Comparisons of retention times between raw files then involves predictions with these raw file-specific models.

Model capabilities

The INFERYS 3.0.0 fragmentation model predicts a set of fragment ions consisting of b-ions, y-ions, water-loss ions ammonia-loss ions, carbon monoxide-loss ions and phosphoric acid-loss ions in charge states 1 to 3. The exact set of ions was predetermined by selecting ions that explain the most experimental intensity in the training data set, hence some ions never or rarely observed in the training data were excluded (e.g. triply charged y1). The fragmentation model is compatible with peptides of length 7 to 30 that can contain carbamidomethylated cysteine residues (fixed), oxidized methionine residues,

phosphorylated serine, threonine or tyrosine residues, Tandem Mass Tag (TMT)-labels, TMTpro-labels and SILAC lysine4, arginine6, lysine8, and arginine10 stable isotope amino acids that were generated by either collision-induced dissociation (resonance-type CID) or higher-energy collisional dissociation (beam-type HCD). The INFERYS 3.0.0 retention time model can predict retention times for the same peptide classes as above. The prediction capabilities of the deep learning models effectively dictate the compatibilities of the CHIMERYS workflow. INFERYS 4.0.0, available with CHIMERYS 4.0.21, extends the capabilities of INFERYS 3.0.0 to acetylated lysine residues and protein N-termini, ubiquitinated lysine residues, unmodified cysteine residues and cysteine residues modified with the PreOmics iST kit.

Data processing and evaluation

Data analysis using various search algorithms

A variety of search engines have been applied in the scope of this study. Unless otherwise noted, default settings were used including trypsin digestion (with proline rule, so cutting after K/R, but not before P), carbamidomethylation of cysteine as fixed modification, as well as methionine oxidation as variable modification. For searches of data from phosphorylated, acetylated or ubiquitinated peptides, phosphorylation of serine, threonine and tyrosine, acetylation of lysine or ubiquitination of lysine were set as variable modifications. Peptide lengths were limited to 7-30 for comparisons between CHIMERYS, DIA-NN and Spectronaut. All searches were performed against the canonical fasta files of the corresponding species or species mix. A contaminants fasta was utilized in all searches to control for contaminations[18]. Entrapment searches were performed as described in detail its own section. For runtime comparisons, the settings of all search engines were harmonized as much as possible. All fasta files used in this study are available via PRIDE (see Data Availability).

CHIMERYS. Searches were performed using CHIMERYS 2.7.9 from PD 3.1.0.622 or 3.1.0.638 (solely used for runtime benchmarks), as well as CHIMERYS 4.0.21 from a pre-release version of PD 3.2 (used for searching the data containing phosphorylated, acetylated or ubiquitinated peptides in Extended Data Figure 4G, as well as for runtime benchmarks) using default settings. For quantification, non-normalized intensities from Minora Feature Detector were used. PSM, precursor, peptide group and protein group IDs were extracted from .pdResult files after the removal of decoys and PSM-, precursor-, peptide group- or protein-level q-value filtering. For ion trap data, an additional TopN Peaks filter node was used before the CHIMERYS node with TopN set to 15 and mass window to 100 Da.

Sequest HT. Searches were performed from PD 3.1.0.622 or PD 3.1.0.638 (solely for runtime benchmarks) using default or harmonized settings. For quantification, non-

normalized intensities from Minora Feature Detector were used. PSM and peptide group IDs were extracted from .pdResult files after the removal of decoys and PSM- or peptide group-level q-value filtering.

MS Amanda. Searches were performed using MS Amanda 3.1.21.532 from PD 3.1.0.638 using default or harmonized settings. For quantification, non-normalized intensities from Minora Feature Detector were used. PSM and peptide group IDs were extracted from .pdResult files after the removal of decoys and PSM- or peptide group-level q-value filtering.

Comet. Searches were performed from PD 3.1.0.622 or PD 3.1.0.638 (solely for runtime benchmarks) with Comet version "2019.01 rev. 1". using default or harmonized settings. For quantification, non-normalized intensities from Minora Feature Detector were used. PSM and peptide group IDs were extracted from .pdResult files after the removal of decoys and PSM- or peptide group-level q-value filtering.

MSFragger. Searches were performed using FragPipe 21.1 with MSFragger 4.0 and Philosopher 5.1.0 using the "Default" workflow or the "WWA" workflow and "DDA+" data type, which considers candidate peptides in the full MS1 isolation window and reports up to the top five PSMs instead of only one. For runtime comparisons, harmonized settings were used. Precursor-level IDs were extracted from ion.tsv files, which were already filtered for decoys and precursor FDR. Peptide group-level IDs were rolled up from precursor-level data, controlled at 1% precursor-level FDR.

MetaMorpheus. Searches were performed using MetaMorpheus 1.0.5 with default or harmonized settings. PSM-level IDs were extracted from AllPSMs.psmtsv files after the removal of decoys and PSM-level q-value filtering. MetaMorpheus does not feature peptide group-level IDs, so these were rolled up from the PSM level, controlled at 1% PSM-level FDR.

MS-GF+. Searches were performed using MS-GF+ 2024.03.2 with default or harmonized settings. The output .mzid file was converted to .tsv, from which PSM-level IDs were extracted after the removal of decoys and PSM-level q-value filtering. MS-GF+ does not feature peptide group-level IDs, so these were rolled up from the PSM level, controlled at 1% PSM-level FDR.

MaxQuant. Searches were performed using MaxQuant 2.4.2.0 or 2.6.5.0 (solely for runtime benchmarks) with default or harmonized settings. Peptide group IDs were extracted from modificationSpecificPeptides.txt files after filtering for decoys, which were already peptide group-level FDR filtered.

DIA-NN. Library-free searches were performed using DIA-NN 1.8.1[19]. Mixed species samples were analyzed with a classic, concatenated, and peptide eFDR approach. Entrapment fasta

files were based on a database for the three species and a database containing contaminants. Detailed information on entrapment database generation can be found under 'Entrapment database construction via mimic'. For all searches, spectral libraries were generated from fasta files using DIA-NN's prediction capabilities. Default settings were used with the following adjustments: Missed cleavages = 0, Maximum number of variable modifications = 2, Quantification strategy = Peak height. 'Ox(M)' was added as a variable modification. Note that 'MBR' is checked per default for library-free searches. The output results from the report.tsv were used. For run-specific precursor filtering, results were filtered for Q.Value ≤ 0.01. Precursor.Quantity was used as precursor quantification values. Fragments for identification were counted considering fragments with Fragment.Quant.Corrected > 0. Fragments for quantification were counted by identifying fragments, for which the sum of Fragment.Quant.Corrected corresponded to Precursor.Quantity. Samples with different species composition were analyzed together.

Spectronaut. Raw files were analyzed using Spectronaut 19 (Biognosys) with a library-free approach (directDIA+). Mixed species samples were analyzed with a classic, concatenated, and peptide eFDR approach. Entrapment fasta files were based on a database for the three species and a database containing contaminants. Detailed information on entrapment database generation can be found under 'Entrapment database construction via mimic'. Default settings were used with the following adjustments: Max Peptide Length = 30, Missed Cleavages = 0, Max Variable Modifications = 2, Quantity Type = Height. Carbamidomethyl (C) was set as a fixed modification, and Oxidation (M) was set as a variable modification. Cross-run normalization was disabled. Samples with different species composition were analyzed together. For run-specific precursor filtering, results were filtered for EG.Qvalue ≤ 0.01. `EG.TotalQuantity (Settings)` was used as precursor quantification values. Fragments for identification were counted considering fragments with F.PeakArea > 1. Fragments for quantification were counted by identifying fragments with F.PeakArea > 1 and F.ExcludedFromQuantification = FALSE.

Post-hoc filtering of Spectronaut output files

We show that Spectronaut claims to confidently identify precursors with no apparent signal in the corresponding MS2 spectra. In order to remove these precursors, an unfiltered, fragment-level export needs to be created from within Spectronaut. Based on this report, fragments with F.PeakArea > 1 and F.ExcludedFromQuantification = FALSE can be identified. In Spectronaut, the desired number of fragments for quantification can be set (under DIA Analysis > Quantification > Interference Correction > MS2 Min). Per default, 'MS2 Min' is three. Hence, in this study, precursors were filtered to only those with at least 'MS2 Min' fragments that have F.PeakArea > 1 and F.ExcludedFromQuantification = FALSE.

Calculation of shared fragment ions

Peptide fragment intensity predictions of PSMs identified by CHIMERYS 2.7.9 were generated using INFERYS 3.0.0. The R package rawrr[20] was used to extract centroided m/z and intensity values from raw files. Different PSMs within the same MS2 scans were tested for shared fragment ions using two methods: directly matching predicted fragment ions (raw file independent) and checking if predicted fragment ions matched to the same raw file peaks with a 20 ppm tolerance. The fraction of shared fragment ions among all fragment ions was determined and analyzed based on amino acid position and 200 m/z bins.

Entrapment database construction via mimic

We generated same-organism entrapment proteins in nine-fold excess relative to the number of target proteins by shuffling sequences at the peptide level using mimic[21] with the command line flags --prepend, --empiric, --replaceI and --mult-factor 9. The goal is to generate nine different entrapment peptides per target peptide, which are isobaric, shuffled versions of it. Entrapment peptides shall have the same peptide N- and C-termini as their target counterpart and shall be as close in amino acid composition to it as possible. Briefly, mimic achieves this by first digesting the target protein database into fully tryptic peptides without missed cleavages (no proline rule, i.e. cleaving proteins after each K/R). Afterwards, the sequence of each unique target peptide is permuted randomly while keeping the termini fixed. In case permutation of a peptide generates a target or previously generated entrapment peptide, shuffling is repeated up to 1,000 times. For this comparison, isoleucine and leucine are considered to be identical amino acids. If no entrapment peptide could be generated after 1,000 rounds of shuffling, amino acids are mutated. Briefly, a random amino acid is mutated to a different one while respecting the amino acid frequencies of the target protein database. Isoleucine and leucine are never mutated when using the --replaceI flag. Notably, it is ensured that target peptides with the same peptide sequence generate the same entrapment peptides. Subsequently, entrapment peptides are assembled to entrapment proteins, which are then either appended to the target database in the case of the classic fasta concatenation approach (classic eFDR) or concatenated to the corresponding target protein sequences – separated by lysine residues – in the sequence concatenation approach (concatenated eFDR). The final digested fasta approach (peptide eFDR) used a peptide-level fasta, which was generated by digesting the classic eFDR fasta file with Protein Digestion Simulator 2.4.7993.32903 before passing it to the search engine (fully tryptic digest with no missed cleavages and no proline rule). Notably, protein-C-terminal peptides cannot be identified with the concatenated eFDR approach and neither can protein N-terminal peptides with methionine excision from entrapment proteins. Mimic is available at

https://github.com/percolator/mimic. A simplified web-version of it is available at https://mimic.msaid.io/.

False discovery rate (FDR) definitions

We adhere to previously established definitions of FDR in the run-specific and global context for any identification level (e.g. precursors, modified peptides, peptides, peptide groups, protein groups)[22]. For example, peptide-level FDR in the run-specific context can answer the question “Which peptides were detected at 1% FDR in this specific LC-MS/MS run?”. Peptide-level FDR in the global context can instead answer the question “Which peptides were detected at 1% FDR in at least one LC-MS/MS run of a given experiment containing multiple LC-MS/MS runs?”.

Calculation of entrapment FDR

We empirically validated the self-reported FDR of CHIMERYS, DIA-NN and Spectronaut using entrapment experiments, also known as double-decoy experiments. The general idea is that most search engines are black boxes that report lists of identifications at self-reported FDR. Entrapment experiments try to empirically validate this self-reported FDR.

Briefly, most if not all search engines try to control the FDR of their identifications at a certain percentage (usually 1%). This means they aim to limit the number of random false targets *FT* among all targets *T*, for example, the number of random false PSMs in a final list of PSMs via the FDR at the PSM level. However, because the identity of random false target identifications is not known *a priori*, search engines model the behavior of random false targets with the help of decoys. Decoys are peptides that resemble target peptides but are assumed to be absent from the sample under investigation. Many if not all search engines generate their own decoy peptides (usually one for every peptide after *in silico* digestion of the target protein database) using one of several approaches (e.g. reversing protein sequences[12]). Experimental MS2 spectra are then compared to theoretical, predicted or library spectra from target and decoy peptides, which results in a score for each PSM that measures how closely they match. In classic target decoy competition, only the top-scoring PSM of each spectrum is retained. Note that CHIMERYS allows for multiple PSMs per MS2 spectrum to account for co-isolated precursors. The PSMs are then sorted in descending order by their score (assuming that a high score is associated with a good match) and q-values are calculated as the cumulative sum of decoys *D* divided by the cumulative sum of targets *T* for each PSM.

Equation (1)

$$FDR \coloneqq \frac{FT}{T} \approx \frac{D}{T}$$

Usually, these q-values are then monotonized by sorting the PSMs by their score in ascending order and calculating the cumulative minimum of the q-values for each PSM to arrive at the final q-values. Removing PSMs with a q-value > 0.01 would filter the list of PSMs to 1% FDR. Notably, as mentioned above, this approach assumes that decoys resemble random false targets and consequently that the score distribution of decoy peptides is identical in shape and magnitude to the score distribution of random false target peptides. If this is not the case, FDR could be over- or underestimated.

Entrapment or double-decoy experiments measure empirical FDR by following very similar principles as the target/decoy approach described above. Before passing the target protein database to the search engine, which then generates its own decoys, so-called entrapment proteins are added to it. Entrapment proteins consist of entrapment peptides, which are very similar to decoy peptides in that they should resemble target peptides but are assumed to be absent from the sample under investigation. However, the search engine does not know which peptide is a 'normal target' peptide and which peptide is an 'entrapment target' peptide. As such, it generates decoy peptides for each 'normal target' and each 'entrapment target' peptide. We will call them 'normal decoys' and 'entrapment decoys' respectively. Experimental MS2 spectra are then compared to theoretical, predicted or library spectra from 'normal target', 'normal decoy', 'entrapment target' and 'entrapment decoy' peptides. Now the search engine calculates its FDR as described above after sorting PSMs based on their score in descending order by dividing the cumulative sum of normal plus entrapment decoy identifications (D_N + D_E) by the cumulative sum of normal plus entrapment target identifications (T_N + T_E), followed by q-value monotonization. In addition, since the identity of entrapments is known to the researcher, an entrapment FDR (eFDR) can be calculated. This is done by sorting PSMs in descending order by their score and calculating entrapment q-values as the cumulative sum of normal decoys plus entrapment targets (D_N + T_E), divided by the cumulative sum of targets plus entrapment targets (T_N + T_E) for each PSM, followed by entrapment q-value monotonization.

The relation of FDR and eFDR is formularized as follows:

Equation (2)
$$FDR \coloneqq \frac{FT}{T} = \frac{FT_N + FT_E}{T_N + T_E} \approx \frac{D_N + D_E}{T_N + T_E} \approx \frac{D_N + T_E}{T_N + T_E} \coloneqq eFDR$$

Assuming false targets and decoys follow the same distribution, the cumulative sum of 'normal false targets' plus 'entrapment false targets' (FT_N + FT_E) can be estimated by the cumulative sum of 'normal decoys' plus 'entrapment decoys' (D_N + D_E). Analogously, assuming that 'entrapment targets' are incorrect identifications and follow the same distribution as 'entrapment decoys', D_E and T_E can be freely exchanged.

For entrapment analyses with DIA-NN and Spectronaut, the formula for calculating eFDR was slightly modified (see Equation (3) below), because their final results violate the assumption that the score distribution of decoy peptides is identical in shape and magnitude to the score distribution of random false target peptides. This is because they perform two searches of the data; one to generate a filtered spectral library and a second one that uses this spectral library to analyze the same data again in a peptide-centric fashion. The filtering of the spectral library is done based on an initial FDR calculation, which will remove a substantial fraction of random false target peptides, causing the distribution of decoy peptides in the second search to not be identical anymore in shape and magnitude to the score distribution of random false target peptides. Hence, the cumulative sum of 'normal decoys' D_N was removed from the eFDR calculation for DIA-NN and Spectronaut. As such, eFDR was calculated by sorting precursors in descending order by their score and calculating entrapment q-values as the cumulative sum of entrapment targets (T_E), divided by the cumulative sum of normal targets plus entrapment targets (T_N + T_E) for each precursor, followed by entrapment q-value monotonization.

Equation (3)

$$eFDR \coloneqq \frac{T_E}{T_N + T_E}$$

Entrapment FDR according to Equation (2) is more conservative than eFDR according to Equation (3) due to the removal of D_N in Equation (3). However, if the population of 'entrapment targets' in the search space is much larger than the population of 'normal decoys' (see below), then the eFDR calculation is mostly influenced by the number of 'entrapment targets' and not by the number of 'normal decoys'. Removing PSMs with an entrapment q-value > 0.01 should also filter the list of PSMs to 1% FDR if the search engine does not have a bias in its scoring function. In other words, if there is no bias, then q-values and entrapment q-values should follow the diagonal if they are plotted against one another in a scatter plot.

The eFDR calculations above point out the need to use entrapment proteins in excess relative to target proteins as search engines generate decoys for each target and each entrapment. In other words, using the same number of entrapments as targets would result in a search space consisting of one part 'normal targets', two parts decoys (one part 'normal decoys' and one part 'entrapment decoys') and one part 'entrapment targets'. Given the nature of random false identifications, they are equally likely to map to false targets, decoys or entrapments. However, we cannot identify them as false matches if they map to false targets. Consequently, the likelihood of a random false identification that we can identify to map to decoys is twice as high (66%) as the likelihood of it mapping to an entrapment (33%) if the number of targets and entrapments were the same. As we increase the number of

entrapments that are added to the target protein database, the likelihood of a random false identification to map to decoys or entrapments becomes more and more similar. At a nine-fold excess of entrapments relative to targets, which we recommend, the likelihood of a random false identification that we can identify to map to a decoy is merely ~6% higher (53%) than the likelihood of it mapping to an entrapment (47%).Entrapments can be added to the target protein database in various different ways (see section 'Entrapment database construction via mimic'). In the case of the concatenated eFDR approach, we had to modify the calculation of eFDR slightly for CHIMERYS, because it is no longer possible to distinguish between 'normal decoys' and 'entrapment decoys'. This is because they are generated internally by CHIMERYS and are both annotated with the target protein identifier, which can be used in the other entrapment approaches to identify 'normal decoys' and 'entrapment decoys'. Hence, for CHIMERYS searches using the concatenated eFDR approach, eFDR calculation was done as described in Equation (3), i.e. in the same way as for DIA-NN and Spectronaut.

Throughout the manuscript, we performed several entrapment analyses for CHIMERYS, DIA-NN and Spectronaut. In Figure 1B and Extended Data Figure 6, we directly compared the self-reported run-specific FDR at a given identification level (e.g. peptides or precursors) to the corresponding eFDR. In Figures 3 and 4, as well as in Extended Data Figure 7, we filtered identifications either at the self-reported run-specific FDR or additionally at the corresponding eFDR. We then compared the characteristics of the identifications that only survive the self-reported FDR or also survive the eFDR (e.g. precision and accuracy of quantification). For CHIMERYS, we used the q-values and SVM scores reported by qvality in PD, which either validated peptide groups in the global context (Figure 1B) or precursors in the run-specific context (all other visualizations). For Spectronaut, we used the EG.Qvalue and the EG.Cscore columns of unfiltered Spectronaut exports, which validated precursors in the run-specific context. For DIA-NN, we used the Q.Value and CScore columns of the main report.tsv, which validated precursors in the run-specific context. Unfortunately, DIA-NN and Spectronaut do not report q-values for decoy identifications and the former always filters its report.tsv to 1% run-specific precursor-level FDR. They do, however, report scores for decoy identifications. In order to convert these scores into q-values for the plots in Extended Data Figure 6, we interpolated the q-values given the relationship of score and q-value for target precursors in each raw file separately.

PRM Analysis in CHIMERYS and Skyline

PRM data were searched using CHIMERYS against a Human Swissport database with default settings. The resulting MS2-based quantities and coefficient traces were extracted from the .pdresult files using custom R scripts. The .raw file was also analyzed in Skyline[23]

22.2 using a Prosit-predicted spectral library[7] as integrated in Skyline. Peak boundaries were manually refined for all precursors and the quantitative values for the area beneath the five most abundant fragment ions was aggregated into a quantitative measure. The Pearson correlation coefficient between quantification values from CHIMERYS and Skyline were calculated using the *cor()* function in R.

Analysis of DI-SPA data with CsoDIAq

DI-SPA data from the original DI-SPA publication[24] were searched with CsoDIAq 2.1.2 using the spectral library published as part of this publication. Additional libraries were generated and searched: a library restricted to precursors predictable with INFERYS, hybrid libraries based on this library, where target or decoy spectra were replaced with INFERYS predictions, a fully predicted library based on the hybrid libraries and a fully predicted library consisting of targets from the hybrid libraries, supplemented with decoys generated by CHIMERYS. All libraries were filtered as described in the CsoDIAq publication[25]. CsoDIAq was compiled and run using default settings as instructed. Raw files were converted into mzXML files using MSConvert 3.0.23121-9c54301[26]. Roll-up to higher levels (e.g. PSM to precursor) and FDR calculation for the peptide group- and protein level was performed manually. For peptide groups, the best representative PSM per peptide group was selected per raw file based on the MaCC score. Peptide groups were constructed by counting the number of unimod occurrences in the modified peptide sequence. FDR was calculated according to Equation (1), followed by q-value monotonization. Protein roll-up and FDR calculation was performed in the same way, but exclusively on unique (i.e. non-shared) peptides, in order to avoid protein grouping differences between search engines. PSMs were filtered at 1% run-specific FDR, peptide groups and protein groups at 1% dataset global peptide group and protein FDR, respectively.

Analysis of DI-SPA data with CHIMERYS

DI-SPA data from the original DI-SPA publication[24] were searched with CHIMERYS 2.7.9 from PD 3.1.0.622. Roll-up to higher levels (e.g. PSM to precursor) and FDR calculation for peptide groups and proteins was performed in the same way as described for CsoDIAq, with the best representative PSM selected based on the SVM score. Quantification was performed by selecting the CHIMERYS coefficient of the best PSM representative, which is equivalent to apex intensity quantification.

KEGG pathway enrichment

KEGG pathway enrichment was performed using Cytoscape 3.10.3[27] with the STRING plugin 2.1.1[28]. Proteins identified by either CHIMERYS or CsoDIAq from raw file 20190405_MCF7_FAIMS_14_1.mzXML were imported into Cytoscape. STRING functional

enrichment was performed using default settings against the human genome as background. Statistically significant KEGG pathways were analyzed in terms of which proteins identified by CHIMERYS and/or CsoDIAq contributed to them. The resulting pie charts were labelled with the STRING enrichment q-value for proteins identified by CHIMERYS.

Comparison of DDA and DIA data using CHIMERYS

Unless otherwise noted, DDA and DIA files were searched with CHIMERYS 2.7.9 from PD 3.1.0.622, using Minora for MS1-level and CHIMERYS for MS2-level quantification, respectively. PSMs were filtered at 1% run-specific FDR, peptide groups and protein groups at 1% dataset global peptide group and protein FDR, respectively. Peptide groups and protein groups with FoundinSamples = 0 were discarded. Protein groups were further filtered for PsmCount > 0 and IsMasterProtein = 0. For quantification of conditions / ratios, at least 2 non-normalized intensity values > 0 were required per condition / in both conditions, respectively (or 3 if stated “all replicates”). For calculating CVs, at least 3 non-normalized intensity values > 0 were required per condition. 2D density estimates were calculated using kde2d from the R package MASS. Entrapment FDR was calculated as described above using the peptide eFDR approach.

Supplementary References

1. Puyvelde, B. V. *et al.* A comprehensive LFQ benchmark dataset on modern day acquisition strategies in proteomics. *Sci. Data* **9**, 126 (2022).

2. Yu, F. *et al.* Analysis of DIA proteomics data using MSFragger-DIA and FragPipe computational platform. *Nat. Commun.* **14**, 4154 (2023).

3. Yang, K. L. *et al.* MSBooster: improving peptide identification rates using deep learning-based features. *Nat. Commun.* **14**, 4539 (2023).

4. Zolg, D. P. *et al.* ProteomeTools: Systematic Characterization of 21 Post-translational Protein Modifications by Liquid Chromatography Tandem Mass Spectrometry (LC-MS/MS) Using Synthetic Peptides*. *Mol. Cell. Proteom.* **17**, 1850–1863 (2018).

5. Fondrie, W. E. & Noble, W. S. mokapot: Fast and Flexible Semisupervised Learning for Peptide Detection. *J. Proteome Res.* **20**, 1966–1971 (2021).

6. Bouwmeester, R., Gabriels, R., Hulstaert, N., Martens, L. & Degroeve, S. DeepLC can predict retention times for peptides that carry as-yet unseen modifications. *Nat. Methods* **18**, 1363–1369 (2021).

7. Gessulat, S. *et al.* Prosit: proteome-wide prediction of peptide tandem mass spectra by deep learning. *Nat. Methods* **16**, 509–518 (2019).

8. Zolg, D. P. *et al.* INFERYS rescoring: Boosting peptide identifications and scoring confidence of database search results. *Rapid Commun. Mass Spectrom.* e9128 (2021) doi:10.1002/rcm.9128.

9. The, M., MacCoss, M. J., Noble, W. S. & Käll, L. Fast and Accurate Protein False Discovery Rates on Large-Scale Proteomics Data Sets with Percolator 3.0. *J Am Soc Mass Spectr* **27**, 1719–1727 (2016).

10. Heil, L. R. *et al.* Evaluating the Performance of the Astral Mass Analyzer for Quantitative Proteomics Using Data-Independent Acquisition. *J. Proteome Res.* **22**, 3290–3300 (2023).

11. Zolg, D. P. *et al.* Building ProteomeTools based on a complete synthetic human proteome. *Nat. Methods* **14**, 259–262 (2017).

12. Tyanova, S., Temu, T. & Cox, J. The MaxQuant computational platform for mass spectrometry-based shotgun proteomics. *Nat. Protoc.* **11**, 2301–2319 (2016).

13. Vaswani, A. *et al.* Attention is All you Need. in *Advances in Neural Information Processing Systems 30 (NIPS 2017)* vol. 30 (Curran Associates, Inc., 2017).

14. Chung, J., Gulcehre, C., Cho, K. & Bengio, Y. Empirical Evaluation of Gated Recurrent Neural Networks on Sequence Modeling. *arXiv* (2014) doi:10.48550/arxiv.1412.3555.

15. Kingma, D. P. & Ba, J. Adam: A Method for Stochastic Optimization. *arXiv* (2014) doi:10.48550/arxiv.1412.6980.

16. Toprak, U. H. *et al.* Conserved Peptide Fragmentation as a Benchmarking Tool for Mass Spectrometers and a Discriminating Feature for Targeted Proteomics*. *Mol. Cell. Proteom.* **13**, 2056–2071 (2014).

17. Li, L., Jamieson, K., DeSalvo, G., Rostamizadeh, A. & Talwalkar, A. Hyperband: A Novel Bandit-Based Approach to Hyperparameter Optimization. *arXiv* (2016) doi:10.48550/arxiv.1603.06560.

18. Frankenfield, A. M., Ni, J., Ahmed, M. & Hao, L. Protein Contaminants Matter: Building Universal Protein Contaminant Libraries for DDA and DIA Proteomics. *J. Proteome Res.* **21**, 2104–2113 (2022).

19. Demichev, V., Messner, C. B., Vernardis, S. I., Lilley, K. S. & Ralser, M. DIA-NN: neural networks and interference correction enable deep proteome coverage in high throughput. *Nat. Methods* **17**, 41–44 (2020).

20. Kockmann, T. & Panse, C. The rawrr R Package: Direct Access to Orbitrap Data and Beyond. *J. Proteome Res.* **20**, 2028–2034 (2021).

21. The, M., MacCoss, M. J., Noble, W. S. & Käll, L. Fast and Accurate Protein False Discovery Rates on Large-Scale Proteomics Data Sets with Percolator 3.0. *J. Am. Soc. Mass Spectrom.* **27**, 1719–1727 (2016).

22. Rosenberger, G. *et al.* Statistical control of peptide and protein error rates in large-scale targeted DIA analyses. *Nat. methods* **14**, 921–927 (2017).

23. Pino, L. K. *et al.* The Skyline ecosystem: Informatics for quantitative mass spectrometry proteomics. *Mass Spectrom. Rev.* **39**, 229–244 (2020).

24. Meyer, J. G., Niemi, N. M., Pagliarini, D. J. & Coon, J. J. Quantitative shotgun proteome analysis by direct infusion. *Nat. Methods* **17**, 1222–1228 (2020).

25. Cranney, C. W. & Meyer, J. G. CsoDIAq Software for Direct Infusion Shotgun Proteome Analysis. *Anal. Chem.* **93**, 12312–12319 (2021).

26. Adusumilli, R. & Mallick, P. Proteomics, Methods and Protocols. *Methods Mol. Biol.* **1550**, 339–368 (2017).

27. Shannon, P. *et al.* Cytoscape: A Software Environment for Integrated Models of Biomolecular Interaction Networks. *Genome Res.* **13**, 2498–2504 (2003).

28. Doncheva, N. T., Morris, J. H., Gorodkin, J. & Jensen, L. J. Cytoscape StringApp: Network Analysis and Visualization of Proteomics Data. *J. Proteome Res.* **18**, 623–632 (2019).
