# Supplementary material for: Unifying the analysis of bottom-up proteomics data with CHIMERYS
Source: Nat Methods. 2025 Apr 22;22(5):1017–27. doi: 10.1038/s41592-025-02663-w (PMC12074992; doi:10.1038/s41592-025-02663-w)
Supplement: Supplementary file 2 — Reporting Summary [file 41592_2025_2663_MOESM2_ESM.pdf]

nature portfolio

Corresponding author(s): Martin Frejno & Mathias Wilhelm

Last updated by author(s): Jan 24, 2025

Reporting Summary

Nature Portfolio wishes to improve the reproducibility of the work that we publish. This form provides structure for consistency and transparency in reporting. For further information on Nature Portfolio policies, see our Editorial Policies and the Editorial Policy Checklist.

Statistics

For all statistical analyses, confirm that the following items are present in the figure legend, table legend, main text, or Methods section.

n/a	Confirmed	
☐	☒	The exact sample size (*n*) for each experimental group/condition, given as a discrete number and unit of measurement
☐	☒	A statement on whether measurements were taken from distinct samples or whether the same sample was measured repeatedly
☒	☐	The statistical test(s) used AND whether they are one- or two-sided<br>*Only common tests should be described solely by name; describe more complex techniques in the Methods section.*
☒	☐	A description of all covariates tested
☒	☐	A description of any assumptions or corrections, such as tests of normality and adjustment for multiple comparisons
☒	☐	A full description of the statistical parameters including central tendency (e.g. means) or other basic estimates (e.g. regression coefficient) AND variation (e.g. standard deviation) or associated estimates of uncertainty (e.g. confidence intervals)
☒	☐	For null hypothesis testing, the test statistic (e.g. *F*, *t*, *r*) with confidence intervals, effect sizes, degrees of freedom and *P* value noted<br>*Give P values as exact values whenever suitable.*
☒	☐	For Bayesian analysis, information on the choice of priors and Markov chain Monte Carlo settings
☒	☐	For hierarchical and complex designs, identification of the appropriate level for tests and full reporting of outcomes
☐	☒	Estimates of effect sizes (e.g. Cohen's *d*, Pearson's *r*), indicating how they were calculated

Our web collection on statistics for biologists contains articles on many of the points above.

Software and code

Policy information about availability of computer code

Data collection	The specific software versions used for LC-MS/MS raw data acquisition is available as meta information in the corresponding raw data.
Data analysis	Raw mass spectrometry data was analyzed with CHIMERYS 2.7.9 from PD 3.1.0.622 or PD 3.1.0.638 using INFERYS 3.0.0, CHIMERYS 4.0.21 from a pre-release version of PD 3.2 using INFERYS 4.0.0, Sequest HT as available in PD 3.1.0.622 or PD 3.1.0.638, MS Amanda 3.1.21.532 from PD 3.1.0.638, Comet 2019.01 rev. 1 as available in PD 3.1.0.622 or 3.1.0.638, MSFragger 4.0 with Philosopher 5.1.0 from FragPipe 21.1 ("Default" and "WWA" workflow with "DDA+" data type), MetaMorpheus 1.0.5, MS-GF+ 2024.03.2, MaxQuant 2.4.2.0 or 2.6.5.0, DIA-NN 1.8.1, Spectronaut 19, Skyline 22.2 and CsoDIAq 2.1.2. Raw files were converted into mzXML files using MSConvert 3.0.23121-9c54301. In silico digest of fasta files was performed using Protein Digestion Simulator 2.4.7993.32903. Models were built and trained with Tensorflow 2.11.1. The Mokapot version used in this study is available on GitHub (https://github.com/wfondrie/mokapot/). The modifications to the mimic entrapment database generator are available on GitHub (https://github.com/percolator/mimic/). A web-version of the mimic tool can be found at https://mimic.msaid.io/. A demo version of Proteome Discoverer and CHIMERYS can be requested at https://www.msaid.de/chimerys-demo or by contacting the corresponding authors. The custom R scripts used for data analysis are available on GitHub (https://github.com/msaid-de/chimerys-manuscript). KEGG pathway enrichment was performed using Cytoscape 3.10.3 with the STRING plugin 2.1.1.

For manuscripts utilizing custom algorithms or software that are central to the research but not yet described in published literature, software must be made available to editors and reviewers. We strongly encourage code deposition in a community repository (e.g. GitHub). See the Nature Portfolio guidelines for submitting code & software for further information.

Data

Policy information about availability of data

All manuscripts must include a data availability statement. This statement should provide the following information, where applicable:

- Accession codes, unique identifiers, or web links for publicly available datasets
- A description of any restrictions on data availability
- For clinical datasets or third party data, please ensure that the statement adheres to our policy

Data Availability

External raw data

The following external data were downloaded from PRIDE or MassIVE and processed with the respective search engines. In brief, body fluid data from Bian et al., 2020 (PXD015087), secretome data from Tüshaus et al., 2020 (PXD018171), Arabidopsis and Halobacterium data from Müller et al., 2020 (PXD014877), phosphorylation data from Frejno et al., 2020 (PXD013615), acetylation and ubiquitination data from Zecha et al., 2022 (PXD023218), triple species mix as well as HeLa data from the LFQBench-type dataset by Van Puyvelde et al., 2022 (PXD028735), Orbitrap Astral data extracted from Gutzman et al., 2024 (PXD046453) and DI-SPA data from Meyer et al. (MSV000085156). Notably, peptides containing methionine residues were excluded from all analyses of the LFQBench-type dataset, since raw files might show differential oxidation. The same applies to the Orbitrap Astral data from PXD046453. For the LFQBench-type dataset, technical replicates were analyzed (see Supplementary Table 3). All other replicates are biological replicates. An itemized mapping of external data processed as part of this study to their source is available in Supplementary Table 3.

Internal raw data

The following datasets were generated in house: FFPE (biological replicates), gradient comparison, wwDDA, instrument generations and PRM data. An overview of the files generated is provided in Supplementary Table 4. The generated mass spectrometric raw and search data of internal datasets from this study are available via PRIDE with the dataset identifier PXD053241.

Fasta files

All fasta files used in this study are available via PRIDE with the dataset identifier PXD053241.

Source Data

All Source and Supplementary Data files required to reproduce this study are available via PRIDE with the dataset identifier PXD053241.

Human research participants

Policy information about studies involving human research participants and Sex and Gender in Research.

Reporting on sex and gender	n/a
Population characteristics	n/a
Recruitment	n/a
Ethics oversight	n/a

Note that full information on the approval of the study protocol must also be provided in the manuscript.

Field-specific reporting

Please select the one below that is the best fit for your research. If you are not sure, read the appropriate sections before making your selection.

☒ Life sciences ☐ Behavioural & social sciences ☐ Ecological, evolutionary & environmental sciences

For a reference copy of the document with all sections, see nature.com/documents/nr-reporting-summary-flat.pdf

Life sciences study design

All studies must disclose on these points even when the disclosure is negative.

Sample size	No sample size calculation was performed. The main focus of this study is to introduce CHIMERYS and compare it to other software packages, which could be done on individual samples.
Data exclusions	Peptides containing methionine residues were excluded from all analyses of the LFQBench-type dataset, since raw files might show differential oxidation. The same applies to the Orbitrap Astral data from PXD046453.

Replication	From publicly available datasets, three replicates were analyzed to assess data variability. The number of replicates is indicated in the corresponding figure legends. All replication attempts for data generated in house were successful.
Randomization	Randomization was not applicable to this study since no biological conditions were compared (e.g. treatment versus control).
Blinding	Blinding was not applicable to this study since no biological conditions were compared (e.g. treatment versus control).

Reporting for specific materials, systems and methods

We require information from authors about some types of materials, experimental systems and methods used in many studies. Here, indicate whether each material, system or method listed is relevant to your study. If you are not sure if a list item applies to your research, read the appropriate section before selecting a response.

Materials & experimental systems

n/a	Involved in the study
☒	☐ Antibodies
☐	☒ Eukaryotic cell lines
☒	☐ Palaeontology and archaeology
☒	☐ Animals and other organisms
☒	☐ Clinical data
☒	☐ Dual use research of concern

Methods

n/a	Involved in the study
☒	☐ ChIP-seq
☒	☐ Flow cytometry
☒	☐ MRI-based neuroimaging

Eukaryotic cell lines

Policy information about cell lines and Sex and Gender in Research

Cell line source(s)	HeLa cells were sourced from ATCC (CCL-2).
Authentication	None of the cell lines used were authenticated. No biological conclusions were drawn based on the data.
Mycoplasma contamination	Cell lines were not tested for mycoplasma contamination. No biological conclusions were drawn based on the data.
Commonly misidentified lines (See ICLAC register)	The DI-SPA data from Meyer et al. (MSV000085156) used MCF7 cells. No biological conclusions were drawn based on the data.
